# Supplementary material for: Businessmen-driven village governance: A viable path for rural collective economic development?
Source: PLoS One. 2026 Feb 13;21(2):e0342687. doi: 10.1371/journal.pone.0342687 (PMC12904470; doi:10.1371/journal.pone.0342687)
Supplement: Attachment — Submitted filename: Review Rep_Round 2_Rural Collective Economic.pdf [file pone.0342687.s004.pdf]

Review report for the paper "Businessmen-Driven Village Governance: a Viable Path for Rural Collective Economic Development?"

Comments

The study addresses a current and relevant phenomenon in the Chinese economy: the involvement of entrepreneurs in the management of rural villages and its effect on the local economy. Compared to previous literature, which tended to focus on the effects of institutional reforms or factors of production, the approach used here is novel. The proposal to consider 'businessmen-driven village governance' (BVG) as a distinct category from 'rich-led governance' is conceptually clear and contributes to the academic debate on human capital and rural governance in China. The empirical analysis uses a representative rural microdata set covering the period from 2021 to 2023. A bidirectional fixed effects model is applied to control for temporal and spatial heterogeneity, and robustness tests are included.

The text is clear and well-structured, and is written in a coherent manner. The methodology is correctly detailed, and the results and discussion of policy implications are consistent.

That said, several aspects should be reviewed. The central issue is the theoretical framework used, as outlined below.

• On line 144 of page 7, it states that the frame of reference will be **endogenous** growth theory and cites Solow's seminal work (Solow, 1956). There are two issues with this approach. Firstly, the model is **exogenous** (long-term economic growth is explained by exogenous technological progress). Secondly, the model does **not distinguish between physical and human capital**. Given this analytical approach, Solow's model does not seem to be the most suitable for this analysis.

• In light of the above, Section 2, particularly 2.1, should be rewritten and the neoclassical growth model of Mankiw, Romer and Weil should be considered :

Mankiw, N. G., Romer, D., & Weil, D. N. (1992). A contribution to the empirics of economic growth. *The quarterly journal of economics*, *107*(2), 407-437.

Although this is an exogenous growth model, it introduces human capital.

• Notably, the findings suggest a tension between efficiency and participation, with democratic consultation negatively moderating the economic effect of the BVG. A deeper discussion of the potential risks of elite capture and the political implications of reducing democratic deliberation in the name of economic efficiency would be beneficial.

review report revised manuscript

The revised document satisfactorily answers all questions and addresses all concerns. The quality of the document has improved substantially and I recommend its publication.
