# Supplementary material for: Businessmen-driven village governance: A viable path for rural collective economic development?
Source: PLoS One. 2026 Feb 13;21(2):e0342687. doi: 10.1371/journal.pone.0342687 (PMC12904470; doi:10.1371/journal.pone.0342687)
Supplement: Attachment — Submitted filename: PLOS ONE Review Report Hassan Tawakol A. Fadol.docx [file pone.0342687.s001.docx]

# Peer Review Report, PLOS ONE

## Manuscript Information

**Title: Businessmen-Driven Village Governance: A Viable Path for Rural Collective Economic Development?**

**Alternative:**

The Economics of Businessmen-Driven Village Governance: Insights from China’s Rural Reform

**Manuscript ID**: PONE-D-25-32951

**Journal**: PLOS ONE

**Reviewer: Hassan Tawakol Ahmed Fadol, Sudan University of Science and Technology, and ORCID:** [**https://orcid.org/0000-0003-4456-9082**](https://orcid.org/0000-0003-4456-9082)

**Abstract:**

This study examines the economic and institutional effects of businessman-driven village governance (BVG) in rural China. Using micro-survey data from 1,028 villages and 18,438 households across 15 provinces (2021–2023), two-way fixed-effects, Propensity Score Matching, and Structural Equation Modeling are applied to identify the mechanisms linking entrepreneurial leadership to rural collective economic development. Results show that BVG significantly increases collective operating income, with former business owners outperforming self-employed cadres. Government project-based support and rural entrepreneurship partially mediate this relationship. While clan networks have no significant moderating role, the democratic consultation system constrains BVG’s efficiency, revealing a trade-off between participatory decision-making and economic performance. Further analysis indicates that BVG enhances rather than erodes farmers’ trust in village cadres. Overall, BVG represents a viable yet institutionally conditional pathway for strengthening China’s rural collective economies and improving local governance effectiveness.

**1. Significance**

The manuscript addresses a timely and policy-relevant issue concerning the role of businessmen-driven village governance (BVG) in promoting rural collective economic development in China. The topic aligns with the national agenda of rural revitalization and contributes to the literature on local governance and institutional economics. The study provides empirical evidence that supports or challenges existing assumptions regarding the economic and social impacts of entrepreneurial leadership in rural China.

## 2. Originality

The paper demonstrates a fair degree of originality. It differentiates between self-employed and business-owner cadres, and examines their distinct impacts on rural collective income, trust, and institutional interactions. Although similar themes exist in Chinese governance literature, the combined use of multi-province micro data and institutional moderation analysis adds novelty. However, the international relevance and theoretical generalization beyond China could be further developed.

## 3. Methodology

The study employs a robust quantitative approach, including two-way fixed-effects regression models, Propensity Score Matching (PSM), and Structural Equation Modeling (SEM). The dataset, drawn from 15 provinces over 2021–2023, is comprehensive and representative. However, the analysis could be strengthened by addressing potential endogeneity or causal inference using instrumental variables or dynamic panel models (e.g., GMM). Further elaboration on the measurement and construction of key mediating variables (government project-based support and rural entrepreneurship activity) is also recommended.

## 4. Presentation and Structure

The manuscript is clearly structured and follows a logical progression from theoretical framing to empirical analysis. Nonetheless, it would benefit from linguistic polishing to reduce redundancy and improve fluency. Figures and tables are informative but require clearer captions and notes. The title could be shortened for precision (e.g., 'Entrepreneurial Village Governance and Rural Collective Development in China'). References should be standardized in accordance with PLOS ONE citation style.

## 5. Weaknesses and Limitations

1. The causal direction between BVG and rural collective income needs stronger identification.
2. The study period (2021–2023) is relatively short for long-term policy implications.
3. The moderating effect of informal institutions (clan networks) is underexplored.
4. Limited comparative discussion with international rural governance experiences.
5. Potential risks of elite capture or conflict of interest are not sufficiently analyzed.

## 6. Recommendation

Based on the overall scientific quality, methodological rigor, and relevance, I recommend **Major Revision**. The paper presents strong potential for publication after addressing methodological issues, expanding theoretical discussion, and improving language and presentation quality.
