# Supplementary material for: Businessmen-driven village governance: A viable path for rural collective economic development?
Source: PLoS One. 2026 Feb 13;21(2):e0342687. doi: 10.1371/journal.pone.0342687 (PMC12904470; doi:10.1371/journal.pone.0342687)
Supplement: Attachment — Submitted filename: Response to Reviewers.docx [file pone.0342687.s003.docx]

**Response letter**

Dear Editors and reviewers,

We sincerely thank you for thoroughly examining our manuscript entitled “***Businessmen-Driven Village Governance: a Viable Path for Rural Collective Economic Development?***” (Manuscript ID: PONE-D-25-32951) and providing very kind and constructive comments. Accordingly, We have carefully proof-read and revised the manuscript substantially to address the comments and further improve the manuscript..

Below, we provide point-by-point responses to reviewers’ comments. The Editor/reviewers’ comments are numbered and laid out below in blue. Our responses are in normal font, and the corresponding revisions in the manuscript are ***italicized***.

# **Responses to Editor**

**Point 1:** Please ensure that your manuscript meets PLOS ONE's style requirements, including those for file naming. The PLOS ONE style templates can be found at [https://journals.plos.org/plosone/s/file?id=wjVg/PLOSOne_formatting_sample_main_body.pdf](https://journals.plos.org/plosone/s/file?id=wjVg/PLOSOne_formatting_sample_main_body.pdf)
**Response 1:**

Thank you for your note. We have carefully reviewed the PLOS ONE style templates and have updated the manuscript and all accompanying files to ensure they now fully adhere to the journal’s formatting and file-naming guidelines.

**Point 2:** PLOS requires an ORCID iD for the corresponding author in Editorial Manager on papers submitted after December 6th, 2016. Please ensure that you have an ORCID iD and that it is validated in Editorial Manager.

**Response2:**

We have completed the required ORCID registration and ensured that my ORCID iD is fully validated in Editorial Manager.

**Point 3:** We suggest you thoroughly copyedit your manuscript for language usage, spelling, and grammar. If you do not know anyone who can help you do this, you may wish to consider employing a professional scientific editing service. Upon resubmission, please provide the following: The name of the colleague or the details of the professional service that edited your manuscript.

**Response3:**

Thank you for your suggestion regarding language refinement. We have thoroughly revised the manuscript to improve language accuracy, including grammar, spelling, and overall clarity.

In addition, the manuscript has been extensively edited for English language by our co-author, Dr. Furong Chen, who has passed the TEM-8 (Test for English Majors-Band 8) and received a one-year CSC-sponsored academic visit at the Leibniz Institute of Agricultural Development in Transition Economies (IAMO) in Germany.

**Point 4:** Thank you for stating the following financial disclosure:
“This work was supported by the Agricultural Science and Technology Innovation Program of the Chinese Academy of Agricultural Sciences [10-IAED-06-2025], and the Project Preliminary Research on the 15th Five-Year Plan for Agricultural and Rural Development: Research on Improving Rural Governance Levels during the 15th Five-Year Plan Period, funded by the Department of Development Planning, Ministry of Agriculture and Rural Affairs of the People's Republic of China.”

Please state what role the funders took in the study. If the funders had no role, please state:
“The funders had no role in study design, data collection and analysis, decision to publish, or preparation of the manuscript.”

If this statement is not correct you must amend it as needed.

Please include this amended Role of Funder statement in your cover letter; we will change the online submission form on your behalf.

**Response 4:**

We confirm that the funders played no role in study design, data collection and analysis, decision to publish, or preparation of the manuscript. As requested, we have included the following statement in the cover letter:

“The funders had no role in study design, data collection and analysis, decision to publish, or preparation of the manuscript.”

**Point 5:** We note that you have indicated that there are restrictions to data sharing for this study. PLOS only allows data to be available upon request if there are legal or ethical restrictions on sharing data publicly.

Before we proceed with your manuscript, please address the following prompts:

a)If there are ethical or legal restrictions on sharing a de-identified data set, please explain them in detail...

b) If there are no restrictions, please upload the minimal anonymized data set necessary to replicate your study findings...

We will update your Data Availability statement on your behalf to reflect the information you provide.

**Response 5:**

Thank you for your comments regarding data sharing. The original survey data used in this study were collected and are managed by the Institute of Agricultural Economics and Development, Chinese Academy of Agricultural Sciences. According to the Institute’s data management regulations, the dataset—which contains sensitive information related to villages, village cadres, and farmers’ households—cannot be publicly shared, even in de-identified form. The restrictions arise from the following considerations:

**Legal and ethical constraints:** The dataset includes sensitive information concerning village governance, cadre characteristics, and households’ economic activities. In accordance with the Data Security Law of the People’s Republic of China, the Personal Information Protection Law, and relevant regulations issued by the Ministry of Agriculture and Rural Affairs, such multi-level microdata are classified as sensitive and must be strictly protected. Public disclosure, even after anonymization, may pose re-identification risks and is therefore legally restricted.

**Institutional data management policies:** The Institute of Agricultural Economics and Development enforces strict authorization procedures for accessing survey data. After confirmation with the Institute’s Data Management Center, we reiterate that datasets containing village-, cadre-, or household-level information may not be distributed externally or uploaded to any public repository, regardless of anonymization, without formal approval under the current policy framework.

**Survey consent and ethical commitments:** During data collection, respondents—including village cadres and farmers—were informed that their information would be kept strictly confidential and used only for scientific research. Public release of the dataset, even in anonymized form, would violate the terms of informed consent and may compromise participant privacy.

In addition, we have uploaded the relevant data usage and disclosure statements in both Chinese and English as an attachment, titled “Data Application & Survey Statement (CN-EN Bilingual)”, for your reference.

**Point 6:** We note that Figure 1 in your submission contain map images which may be copyrighted...

We require you to either (1) present written permission from the copyright holder... or (2) remove the figures from your submission.

**Response 6:**

Thank you for noting this. To avoid any potential copyright concerns, we have removed Figure 1 in the revised manuscript and have carefully reviewed all other figures to ensure full compliance.

**Point 7:** If the reviewer comments include a recommendation to cite specific previously published works...

**Response 7:**

We sincerely appreciate Reviewer 2’s thoughtful and constructive suggestions. Upon careful review, we find the suggested references highly relevant and closely aligned with the focus of our study. The recommended works have now been appropriately integrated into the revised manuscript.

# **Responses to Reviewer 1**

**Point 1:** Lack of rigorous causality testing: Despite the use of panel data, no causality tests such as GMM or instrumental variables were included to verify the causal direction between BVG and collective output.

**Response 1:**

Thank you very much for this valuable comment. We fully agree that endogeneity and causal identification are critical when assessing the effect of businessman-driven village governance (BVG) on rural collective economy. In response, we have incorporated an instrumental variable strategy to strengthen the causal interpretation of our findings.

Specifically, we use the shortest geographical distance between sample villages and the historical origins of China's ten major merchant guilds as an instrumental variable for BVG. This variable theoretically satisfies both conditions of relevance and exogeneity. Firstly, regions historically situated closer to merchant guild origins are likely to have been more deeply influenced by commercial cultural, fostering stronger traditions of private enterprise, market awareness, and a higher prevalence of business experience within local communitiy. This entrenched commercial environment helps cultivate the social foundations for BVG, thereby increasing the probability of village secretaries possessing business backgrounds. Accordingly, this variable are theoretical relevance to BVG. Secondly, the historical location of merchant guilds were primarily determined by geographical conditions, transportation networks, and historical divisions of labour during the Song, Ming, and Qing dynasties. There factors have no direct relation to contemporary rural collective economic outcomes. Thus, it satisfies the exogeneity requirement for instrumental variables. Furthermore, following the methodology of previous studies [73,74], we conducted an exclusion restriction test by including both the instrumental variable and the BVG variable into the model to further examine the validity of our instrument.

In practical terms, the construction of the instrumental variable proceeded in three steps:

1.Data collection. Using AutoNavi Maps, we collected the latitude and longitude coordinates for the locations of village committees in all sample villages, as well as the origins of each merchant guild. Referencing Wang et al.[75], the origins of the merchant guilds are defined as follows: the Shanxi Merchant Guild originates from Pingyao County and Linfen City; the Shaanxi Merchant Guild from Sanyuan County, Jingyang County, and Suide County; the Ningbo Merchant Guild: Yinxian County, Fenghua City, Cixi City, Zhenhai District, Dinghai District, and Xiangshan County; the Shandong Merchant Guild: Zhoucun District; the Guangdong Merchant Guild: Guangzhou City, Chaozhou City, Haiyang City, Chenghai District, Raoping County, and Dapu County; the Fujian Merchant Guild: Quanzhou City, Xiamen City, and Zhangzhou City; the Dongting Merchant Guild: Suzhou City; The Jiangyou merchant guild comprises Nanchang City, Ji'an City, Fuzhou City, and Yichun City; the Longyou merchant guild encompasses Longyou County, Changshan County, Qu County, Kaihua County, and Jiangshan City; the Huizhou merchant guild includes She County, Xiuning County, Wuyuan County, Qimen County, Yi County, and Jixi County.

2. Distance calculation. For each village, we calculated the spherical distance between each sample village and all merchant guild origins using Stata's geodist command, and selected the shortest distance as the instrumental variable.

3. Panel adaptation. Since this distance measure is time-invariant, we followed established practice [76–78] by interacting it with year dummy variables to create a panel-compatible instrument for use in two-stage least squares (2SLS) estimation.

Table 4 presents the results of the instrument validity test. In the first-stage regression, the IV coefficient is significantly negative at the 10% level, indicating that the probability of BVG decreases as the distance from merchant guild origins increases. This demonstrates that the selected instrumental variables satisfy the correlation condition. Concurrently, the K-P LM statistic of 46.338 passes the unidentifiability test, while the K-P WF statistic of 35.683 exceeds the critical value of 16.38 at the 10% significance level. This confirms that the selected instrumental variable does not suffer from unidentifiability or weak instrument issues, thus satisfying the exogeneity condition. In the second-stage regression, the estimated coefficient on BVG remain positive and statistically significant at the 1% level, indicating that after addressing potential reverse causality, BVG still significantly promotes the development of rural collective economy. We further conducted an exclusion restriction test by including both BVG and the instrumental variable in the same model. Results show that the instrumental variable becomes statistically insignificant, whereas the coefficient on BVG remains largely unchanged in both magnitude and significance compared to the baseline estimates. This supports the validity of the exclusion restriction. In summary, the conclusions drawn in this paper are reasonable.

Table 4. Instrument variable test

| Variables | First stage | Second stage | Exclusion restriction test |
| --- | --- | --- | --- |
| BVG |  | 1.778^***^ | 0.958^***^ |
|  |  | (0.591) | （0.310） |
| IV | -0.038^*^ |  | -0.089 |
|  | (0.022) |  | (0.057) |
| Controls | Yes | Yes | Yes |
| Individual fixed effects | Yes | Yes | Yes |
| Time fixed effects | Yes | Yes | Yes |
| Kleibergen-Paap LM | 46.338^***^ | |  |
| Kleibergen-Paap rk Wald F | 35.683 [16.38] | |  |
| N | 946 | | 946 |
| R^2^ |  | | 0.957 |

**Point 2:** The study period is short (2021–2023), which reduces the model's ability to capture long-term dynamics.

**Response 2:**

Thank you very much for this important comment. We fully agree that a three-year observation window limits the model’s ability to capture long-term dynamic changes.

The short study period results from data availability rather than researcher choice. Although the national rural micro-survey used in this study has been conducted annually since 2012, the relevant indicators for measuring businessman-driven village governance (BVG), such as village cadres’ prior business experience, entrepreneurial background, and governance participation, were only introduced in the 2021 questionnaire revision. Therefore, consistent and comparable measurement of BVG across villages is only possible from 2021 onward.

Additionally, while the 2024 survey has been completed, the data are still under processing and have not yet been released to researchers within our institute, and therefore cannot be incorporated into the present analysis. Given these constraints, the period 2021–2023 represents the only continuous time span for which complete, standardized, and fully comparable BVG indicators are available.

In the revised manuscript, we have explicitly clarified this data limitation and added a discussion of its implications for long-term inference.

*Third, the time span of the dataset used in this study is limitied to the years from 2021 to 2023, which constrains the ability to assess the long-term effects and dynamic changes of BVG. Future research could extend the observation period and incorporate qualitative methods, such as in-depth interviews, to further explore the underlying behavioral mechanisms and long-term governance outcomes associated with entrepreneurial cadres. (Line 776-782)*

**Point 3:** Lack of adequate description of the mediating variables, particularly the indicators of "project-based government support" and "rural entrepreneurial activity." Their calculation methods and sources need to be clarified.

**Response 3:**

Thank you for this constructive comment. We agree that the definitions, data sources, and calculation methods of the mediating variables require clearer explanation. In the revised manuscript, we have substantially expanded the measurement descriptions of both mediators as follows:

*Based on the mechanism analysis, we examines two mediating mechanisms: government project-based support and rural entrepreneurship activity. In rural China, the project-based approach is the primary means of fiscal transfer and the allocation of policy resources, reflecting a village’s capacity to obtain external resources and policy support [59]. Previous studies commonly use either the number of government projects undertaken by a village or the total investment in these projects to measure the level of government support. For example, Qian et al. [60] use the number of projects undertaken to capture a village’s access to policy resources, while Han et al. [27] use total project investment to characterize the intensity of government support. Since fiscal funds in rural China are primarily distributed through specific projects, and the scale of these projects varies considerably, this study adopts the total investment of government projects undertaken by a village as the indicator of government support.*

*Rural entrepreneurial activity is an important indicator of regional economic vitality and endogenous development potential, but there is no standardized measurement in the literature. Commonly used indicators include the number of individual businesses, private enterprises, online stores, village-level industrial projects, and the number of people engaged in secondary and tertiary industries [61,62]. Among these, the entrepreneurial activities dominated by individual businesses are characterized by “small scale, large quantity, and frequent entry and exit,” representing the main form of rural entrepreneurship in China [63]. Zhang et al. [64] indicate that the annual number of newly registered individual businesses can effectively capture the intensity of entrepreneurial activity. This approcah aligns with the World Bank Entrepreneurship Database, which also treats the number of individual businesses as a core indicator of entrepreneurial activity and provides standardized definitions. (Line 358-383)*

**Point 4:** Weak comparative discussion with the literature on other developing countries (outside of China), limiting the theoretical contribution to the global context.

**Response 4:**

We sincerely thank the reviewer for this insightful comment. In response, we have substantially revised the Introduction and Contribution sections to strengthen the international comparative perspective and clarify the global relevance of our findings.

First, in the Introduction, we now explicitly frame rural development challenges as issues widely shared across developing countries. We added comparative discussions drawing on international literature that highlights common constraints such as limited governance capacity, resource scarcity, and institutional constraints. We also incorporated comparative references to international studies—such as Martiskainen et al. (grassroots innovation) and Kusio et al. (local leadership in EU rural regions)—to demonstrate that the role of entrepreneurial leaders is not unique to China, but reflects a broader global phenomenon.

Second, we expanded the discussion on human capital and grassroots leadership to situate village cadres within the wider international scholarship on local governance in the Global South. The revised sections highlight that many developing countries similarly emphasize leadership quality and entrepreneurial capacity as key drivers of local economic development.

Third, in the Contribution section, we added a new paragraph clarifying how our findings extend to the global context. Specifically, we emphasize that the mechanisms identified in this study—linking business experience, governance capacity, and collective economic outcomes—offer theoretical insights applicable to countries facing similar governance constraints.

The specific modifications are as follows:

*The countryside serves as a fundamental socioeconomic unit in China and many other nations, supporting the livelihood and economic activities of most rural residents [1–3]. Globally, rapid urbanisation and industrialisation have driven significant economic growth in cities over recent decades, while rural areas, particularly those in developing countries, commonly face shared challenges such as resource scarcity, institutional constraints, and limited governance capacity [4,5]. Identifying effective ways to stimulate rural economic development vitality and enhance farmers' welfare has become a major issue of universal concern across nations [6–8].*

*Existing research on rural economic development pathways can be broadly divided into two strands. One perspective emphasises the foundational role of institutional factors, arguing that institutional innovation is an essential prerequisite for revitalising rural development and optimising resource allocation*

*[9–11]. The other highlights the driving force of factor accumulation and allocation efficiency, underscoring the pivotal influence of resources, capital, technology, and human capital on rural economic development [12–15]. Among these, human capital—considered the most central and dynamic factor in socio-economic development [16]—has received growing attention for its vital role in driving rural economic growth, raising household incomes, and enhancing rural governance [17–20]. For instance, Martiskainen et al. [21] note that effective community leaders typically leverage forms of tacit knowledge, including networking, resource integration, and talent identification, which plays a pivotal role in grassroots innovation. Kusio et al. [22], using data from multiple EU countries, find that the absence of local leaders capable of uniting communities around shared objectives constitutes a significant constraint on rural development. Consequently, enhancing the quality of rural human capital, particularly by strengthening grassroots leadership capabilities, has become a key priority for many nations in advancing rural revitalisation.*

*As one of the world's most populous developing nations, China……（Line 31-153）*

*Overall, this study offers several important contributions. Empirically……Practically, the findings offer policy-relevant insights for promoting rural economic revitalization in developing countries. Specifically, they highlight the importance of systematic investment in rural human capital and the institutionalization of mechanisms to identify and cultivate local talents who combine business experience with governance skills, thereby enabling them to play a more prominent role in resource integration, market expansion, and project management.（Line 745-766）*

**Point 5:** Institutional analysis needs to be expanded: The relationship between family clans and economic governance deserves deeper justification, especially in light of the results of statistical insignificance.

**Response 5:**

We thank the reviewer for this insightful comment. We agree that the institutional explanation regarding clan networks requires deeper elaboration, especially given the statistically insignificant moderating effect observed in our empirical results. In the revised manuscript, we have substantially expanded the theoretical discussion. The main additions are summarized as follows:

*As an informal institution, clan networks do not significantly moderate the positive impact of merchant governance on rural collective economic income. This finding is not entirely consistent with the initial expectation. Several explanations may account for this result. First, clan culture, as a prominent aspect of Chinese tradition, has historically contributed to socioeconomic development. Nevertheless, most contemporary policies are designed to suppress clan influences, given that clans have been associated with intense social conflicts, persistent tensions between officials and villagers, and potential risks to social stability [53]. Second, rapid urbanization and the disintegration of traditional village structures have substantially weakened the internal cohesion, mobilization capacity, and internal norms of clan cohesion. Even where surname-based clans remain visible, their actual social influence and ability to broker collective action have declined markedly [82]. Consequently, clans now exert limited constraints on the economic management behaviors of village cadres. Furthermore, the modernization of China’s rural governance system has strengthened in recent years. Under the leadership of village Party organizations, grassroots governance has become more standardized and institutionalized. With the widespread implementation of formal institutions—such as the “three resources” supervision platforms, financial disclosure mechanisms, and village affairs supervision committees—rural economic affairs have increasingly been incorporated into a formalized governance framework. The strengthening of formal institutions has objectively further reduced the space for clan networks to intervene in rural economic resource allocation and oversight, limiting their potential moderating effect in the context of merchant governance. In other words, as a declining informal institution, the normative influence of clan networks has been increasingly marginalized within the modern rural governance structure, resulting in an insignificant moderating effect. （Line 660-684）*

**Point 6:**The language of the paper is generally good but needs language revision to reduce repetition and refine some terminology (e.g., “businessman-type village cadres” could be shortened to “entrepreneurial cadres”)

**Response 6:**

Thank you for your helpful suggestion regarding language refinement. We have carefully reviewed the manuscript to reduce repetition and improve overall clarity. Specifically, we have standardized the terminology by replacing the phrase “businessman-type village cadres” with the more concise and academically appropriate term “entrepreneurial cadres” throughout the manuscript.

# **Response to Reviewer 2**

**Point 1:** The study addresses a current and relevant phenomenon in the Chinese economy: the involvement of entrepreneurs in the management of rural villages and its effect on the local economy. Compared to previous literature, which tended to focus on institutional reforms or factors of production, the approach used here is novel. The proposal to consider 'businessmen-driven village governance' (BVG) as a distinct category from 'rich-led governance' is conceptually clear and contributes to the academic debate on human capital and rural governance in China. The empirical analysis uses a representative rural microdata set covering the period from 2021 to 2023. A bidirectional fixed effects model is applied to control for temporal and spatial heterogeneity, and robustness tests are included. The text is clear and well-structured, and is written in a coherent manner. The methodology is correctly detailed, and the results and discussion of policy implications are consistent.

**Response 1:**

We sincerely appreciate the reviewer’s positive evaluation and encouraging comments. Your feedback has strengthened our confidence in the research design and the contribution of this study. In the revised manuscript, we have further refined and improved the relevant sections in accordance with your suggestions.

**Point 2:** That said, several aspects should be reviewed. The central issue is the theoretical framework used, as outlined below. There are two issues with this approach. Firstly, the model is exogenous (long-term economic growth is explained by exogenous technological progress). Secondly, the model does not distinguish between physical and human capital. Given this analytical approach, Solow's model does not seem to be the most suitable for this analysis. In light of the above, Section 2, particularly 2.1, should be rewritten and the neoclassical growth model of Mankiw, Romer and Weil should be considered: Although this is an exogenous growth model, it introduces human capital.

**Response 2:**

We sincerely appreciate the reviewer’s careful reading and insightful theoretical comments. Thanks for noting the limitations of the classical Solow model—specifically its exogenous treatment of technological progress and its lack of distinction between physical and human capital. We fully agree that these characteristics make the original Solow framework less suitable for analyzing the mechanisms of businessman-driven village governance.

In accordance with your recommendation, we have substantially revised Section 2, particularly Section 2.1. The revised version now incorporates the Mankiw–Romer–Weil (MRW) neoclassical growth model, which extends the Solow model by explicitly including human capital as a separate production factor. This modification better aligns with our conceptualization of entrepreneurial cadres and their influence on rural economic development through human capital, governance capacity, and institutional upgrading.

The revised content is presented below:

*The development of the rural collective economy is fundamentally a matter of economic growth. To analyze the influence of BVG, we therefore draw on classical economic growth theory. According to the neoclassical growth framework, human capital represents an accumulation factor of equal importance to physical capital in economic expansion. Following Mankiw et al. [41], we formulate an extended Cobb–Douglas production function incorporating physical capital, human capital, and labour.*

$$\text{Y}\left( \text{t} \right)\text{=}\text{K}\left( \text{t} \right)^{\text{α}}\text{H}\left( \text{t} \right)^{\text{β}}\left[ \text{A}\left( \text{t} \right)\text{L}\left( \text{t} \right) \right]^{\text{1}\text{−}\text{α}\text{−}\text{β}}$$

*Where Y represents rural collective economy development, K denotes physical capital, H is the stock of human capital, and L denotes labour, 0<α<1,0<β<1, and α+β<1.*

*We assume that BVG can enhance the overall human capital stock through entrepreneurial experience and market-oriented capabilities. For analytical purposes, let C denote the entrepreneurial aptitude coefficient. The human capital stock of villages led by entrepreneurial cadres is then:*

$$\text{H}_{\text{1}}\text{=}\left( \text{1}\text{+}\text{λ} \right)\text{H}_{\text{0}}$$

*The human capital stock of villages led by non-entrepreneurial cadres is* $\text{H}_{\text{0}}$

*With A, K, and L held constant, the production function expression for villages with village cadres possessing business experience is:*

$$\text{Y}_{\text{1}}\text{=}\text{A}^{\text{1}\text{−}\text{α}\text{−}\text{β}}\text{K}^{\text{α}}\left[ \left( \text{1}\text{+}\text{λ} \right)\text{H}_{\text{0}} \right]^{\text{β}}\text{L}^{\text{1}\text{−}\text{α}\text{−}\text{β}}$$

*The production function for villages led by non-entrepreneurial cadres is:*

$$\text{Y}_{\text{0}}\text{=}\text{A}^{\text{1}\text{−}\text{α}\text{−}\text{β}}\text{K}^{\text{α}}\text{H}_{\text{0}}^{\text{β}}\text{L}^{\text{1}\text{−}\text{α}\text{−}\text{β}}$$

*Assuming a state of perfect competition in the market, the marginal product of labour (MPL) for cadres can be expressed respectively as:*

$$\text{MPL}_{\text{1}}\text{=}\left( \text{1}\text{−}\text{α}\text{−}\text{β} \right)\text{A}^{\text{1}\text{−}\text{α}\text{−}\text{β}}\text{K}^{\text{α}}\left[ \left( \text{1}\text{+}\text{λ} \right)\text{H}_{\text{0}} \right]^{\text{β}}\text{L}^{\text{−}\left( \text{α}\text{+}\text{β} \right)}$$

$$\text{MPL}_{\text{0}}\text{=}\left( \text{1}\text{−}\text{α}\text{−}\text{β} \right)\text{A}^{\text{1}\text{−}\text{α}\text{−}\text{β}}\text{K}^{\text{α}}\text{H}_{\text{0}}^{\text{β}}\text{L}^{\text{−}\left( \text{α}\text{+}\text{β} \right)}$$

*By means of relative values, one may further deduce the contribution effect of the entrepreneurial capabilities of cadres on output as follows:*

$$\text{C}\text{=}\frac{\text{MPL}_{\text{1}}}{\text{MPL}_{\text{0}}}\text{=}\left( \text{1}\text{+}\text{λ} \right)^{\text{β}}\text{>}\text{1}$$

*When λ>1, C>1. Accordingly, the partial derivative with respect to entrepreneurial aptitude is expressed as:*

$$\text{Q=}\frac{\text{∂}\text{C}}{\text{∂}\text{λ}}\text{=}\text{β}\left( \text{1}\text{+}\text{λ} \right)^{\text{β}\text{−}\text{1}}\text{>}\text{0}$$

*where Q represents the contribution coefficient of entrepreneurial aptitude to output performance. The findings indicate that higher entrepreneurial aptitude can significantly enhance economic performance, suggesting that entrepreneurial cadres exert a stronger positive effect on the development of the rural collective economy. Imprinting theory posits that unique experiences acquired during specific stages of socialization exert enduring influences on subsequent behaviour [42]. Grounded in resource dependence theory, rural development heavily relies on the acquisition and integration efficiency of external resources [43]. Within these theoretical framework, entrepreneurial cadres typically possess distinct advantages in market expertise and interpersonal networks. These enable them to identify, secure, and integrate external resources more effectively, thereby enhancing resource allocation efficiency [44]. Existing research indicates that such cadres often hold dual identities as both political and economic elites[28], with their governance practices exhibiting a pronounced orientation towards economic performance and efficiency [27]. Concurrently, their entrepreneurial background endows them with cognitive capabilities to discern development opportunities and innovative pathways, enabling them to better navigate market risks and uncertainties within rural governance practices. This, in turn, fosters the sustained development of the rural collective economy. Accordingly, we propose the following hypothesis:*

*H1: BVG contributes to the development of the rural collective economy.（Line 157-200）*

**Point 3:** Notably, the findings suggest a tension between efficiency and participation, with democratic consultation negatively moderating the economic effect of the BVG. A deeper discussion of the potential risks of elite capture and the political implications of reducing democratic deliberation in the name of economic efficiency would be beneficial.

**Response 3:**

Thank you for this thoughtful and important comment. We fully agree that the tension between economic efficiency and participatory governance is a central issue in rural political economy, and that our discussion should further address the risks of elite capture and the broader political implications of weakening democratic deliberation. In response, we have substantially expanded the relevant section in the revised manuscript.

Specifically, we provide a deeper theoretical explanation of why democratic consultation—an essential component of China’s formal rural governance system—may negatively moderate the economic effect of BVG. We also discuss the governance risks associated with overly efficiency-oriented reforms, particularly the possibility that the “strongman-driven” nature of BVG could evolve into exclusive power structures, “oligarchic governance”, or elite capture if not properly constrained.

The expanded discussion emphasizes that, despite the potential economic gains from BVG, the checks and balances provided by democratic consultation mechanisms remain indispensable. We highlight that safeguarding procedural fairness, preventing power concentration, and protecting villagers’ rights may require accepting a certain degree of economic inefficiency—an essential trade-off in maintaining governance equity and preventing elite capture.

The specific modifications are as follows:

*It is worth noting that the democratic consultation mechanism—a core component of the formal institutional system—significantly constrains the economic effectiveness of BVG. As a key form of villagers’ self-governance, the democratic consultation mechanism aims to broaden farmers’ participation in village affairs, including decision-making, management, and oversight. By emphasizing democratic principles in collective action, it promotes fairness in the distribution of collective interests [83,84]. In villages with a higher degree of democratization, governance procedures tend to be more complex, which may limit the efficiency advantages typically associated with business-village governance cadres. This phenomenon reflects a deeper tension between market-oriented logic and governance-oriented logic in rural governance, particularly when balancing efficiency with democratic objectives. However, despite the potential of BVG to integrate resources and stimulate the development of rural collective economies, this result should be interpreted with caution. It is especially important to avoid weakening democratic consultation in the name of economic efficiency. The concern arises from the inherent “strongman-driven” nature of the BVG: while it may raise decision-making efficiency, it can also give rise to exclusive power structures, potentially leading to “oligarchic governance” or elite capture, ultimately undermining villagers’ collective interests [85]. A case study of Lanjing Village in Sichuan Province illustrates that in village-level enterprises established under BVG leadership, profit distribution tends to favor returning entrepreneurs who serve as initial investors, while ordinary villagers receive only a limited share—thereby exacerbating socioeconomic stratification within rural communities [86]. These insight highlight the need to balance economic benefits with governance risks. Therefore, maintaining the checks and balances afforded by democratic consultation institutions is essential, even if this entails some economic trade-offs. Sacrificing a degree of efficiency in exchange for social equity and governance fairness is a necessary compromise to safeguard villagers’ fundamental interests and prevent elite capture.(Line 685-712)*

Once again, we sincerely thank the editors and reviewers for their valuable feedback and constructive comments, which have greatly improved the quality of this manuscript. We would be grateful for the opportunity to have it considered for publication in PLOS ONE.

**Newly Added References**

1. Abawa A. Entrepreneurial competency in agriculture: Is it a panacea for the problem of farmers’ welfare? J Agribus Dev Emerg Econ. 2024;14:60–76. <https://doi.org/10.1108/JADEE-01-2022-0006>
2. Bebbington A, Dharmawan L, Fahmi E, Guggenheim S. Local capacity, village governance, and the political economy of rural development in Indonesia. World Dev. 2006;34:1958–76. <https://doi.org/10.1016/j.worlddev.2005.11.025>
3. Bernard T, Collion M-H, de Janvry A, Rondot P, Sadoulet E. Do village organizations make a difference in African rural development? A study for Senegal and Burkina Faso. World Dev. 2008;36:2188–204. <https://doi.org/10.1016/j.worlddev.2007.10.010>
4. Cao X, Deng M, Li H. How does e-commerce city pilot improve green total factor productivity? Evidence from 230 cities in China. J Environ Manage. 2021;289:112520. <https://doi.org/10.1016/j.jenvman.2021.112520>
5. Cui B, Ma K. Can cooperatives become an effective carrier for rural governance in China? On the unexpected function of cooperatives. Chin Rural Econ. 2022;41–58.
6. Dansou HD, Carrier M. Decentralization, institutional innovation and governance of inter-territorial relations: A view from Benin. Cities. 2023;133:104115. <https://doi.org/10.1016/j.cities.2022.104115>
7. Ding B. The effectiveness of rural collective economy and rural governance under the background of rural revitalization: Field survey based on four villages in southern Anhui. J Nanjing Agric Univ (Soc Sci Ed). 2020;20:53–61.
8. Dinkelman T. The effects of rural electrification on employment: New evidence from South Africa. Am Econ Rev. 2011;101:3078–108. <https://doi.org/10.1257/aer.101.7.3078>
9. Du X, Weng J, Zeng Q, Pei H. Culture, marketization, and owner–manager agency costs: A case of merchant guild culture in China. J Bus Ethics. 2017;143:353–86. <https://doi.org/10.1007/s10551-015-2765-3>
10. Duflo E, Pande R. Dams. Q J Econ. 2007;122:601–46. https://doi.org/10.1162/qjec.122.2.601
11. Faber B. Trade integration, market size, and industrialization: Evidence from China’s national trunk highway system. Rev Econ Stud. 2014;81:1046–70. <https://doi.org/10.1093/restud/rdu010>
12. Futemma C, De Castro F, Brondizio ES. Farmers and social innovations in rural development: Collaborative arrangements in eastern Brazilian Amazon. Land Use Policy. 2020;99:104999. <https://doi.org/10.1016/j.landusepol.2020.104999>
13. Gittins P, McElwee G. Constrained rural entrepreneurship: Upland farmer responses to the socio-political challenges in England’s beef and sheep sector. J Rural Stud. 2023;104:103141. <https://doi.org/10.1016/j.jrurstud.2023.103141>
14. Graser M, Bonatti M, Eufemia L, Morales H, Lana M, Löhr K, et al. Peacebuilding in rural Colombia—A collective perception of the integrated rural reform (IRR) in the department of Caquetá (Amazon). Land. 2020;9:36. <https://doi.org/10.3390/land9020036>
15. Guo Y, Zhang K, Xu J. Coupling coordination degree of entrepreneurship and green development with digital transformation in China. Digit Econ Sustain Dev. 2025;3:15. <https://doi.org/10.1007/s44265-025-00062-9>
16. Haji K. E-commerce development in rural and remote areas of BRICS countries. J Integr Agric. 2021;20:979–97. <https://doi.org/10.1016/S2095-3119(20)63451-7>
17. Hoque MR. The impact of the ICT4D project on sustainable rural development using a capability approach: Evidence from Bangladesh. Technol Soc. 2020;61:101254. <https://doi.org/10.1016/j.techsoc.2020.101254>
18. Koutridi E, Christopoulou O. The importance of integrating smart farming technologies into rural policies (aiming at sustainable rural development): Stakeholders’ views. Smart Agric Technol. 2023;4:100206. <https://doi.org/10.1016/j.atech.2023.100206>
19. Kusio T, Kudełko J, Borges A, Delic A, Stroila I. Are there any differences in rural development challenges within European countries? Social and economic contexts from EU rural leaders. Int Food Agribus Manag Rev. 2022;25:737–56. <https://doi.org/10.22434/IFAMR2021.0147>
20. Li N, Tang L, Che X, Shi X, Ma X. Does the democratization level of village governance affect perceptions of security and integrity of land rights? An analysis from the perspective of social network abundance. J Rural Stud. 2022;94:305–18. <https://doi.org/10.1016/j.jrurstud.2022.06.013>
21. Liang C, Zhou Y. Unlocking rural vitality: Assessing the multidimensional impacts of land consolidation on rural comprehensive development capacity in post-poverty China. J Rural Stud. 2025;120:103870. <https://doi.org/10.1016/j.jrurstud.2025.103870>
22. Lowery B, Dagevos J, Chuenpagdee R, Vodden K. Storytelling for sustainable development in rural communities: An alternative approach. Sustainable Dev. 2020;28:1813–26. <https://doi.org/10.1002/sd.2124>
23. Mankiw NG, Romer D, Weil DN. A contribution to the empirics of economic growth. Q J Econ. 1992;107:407–37. <https://doi.org/10.2307/2118477>
24. Martiskainen M. The role of community leadership in the development of grassroots innovations. Environ Innov Soc Transit. 2017;22:78–89. <https://doi.org/10.1016/j.eist.2016.05.002>
25. Muñoz P, Kimmitt J. Social mission as competitive advantage: A configurational analysis of the strategic conditions of social entrepreneurship. J Bus Res. 2019;101:854–61. <https://doi.org/10.1016/j.jbusres.2018.11.044>
26. Naron V. Entrepreneurial spirit is a panacea for rural development. Unpublished manuscript; 2014. <https://doi.org/10.13140/RG.2.2.19686.73286>
27. Pfeffer J, Salancik G. External control of organizations: Resource dependence perspective. In: Organizational Behavior 2. London: Routledge; 2006.
28. Rao V, Ananthpur K, Malik K. The anatomy of failure: An ethnography of a randomized trial to deepen democracy in rural India. World Dev. 2017;99:481–97. <https://doi.org/10.1016/j.worlddev.2017.05.037>
29. Rodríguez-Pose A, Hardy D. Addressing poverty and inequality in the rural economy from a global perspective. Appl Geogr. 2015;61:11–23. <https://doi.org/10.1016/j.apgeog.2015.02.005>
30. Sabet NS, Khaksar S. The performance of local government, social capital and participation of villagers in sustainable rural development. Soc Sci J. 2024. Available from: <https://www.tandfonline.com/doi/abs/10.1080/03623319.2020.1782649>
31. Shah M, Steinberg BM. Workfare and human capital investment: Evidence from India. J Hum Resour. 2021;56:380–405. <https://doi.org/10.3368/jhr.56.2.1117-9201R2>
32. Tshidzumba RP, Chirwa PW. Forest-based land reform partnerships in rural development and the sustenance of timber markets: Learning from two South African cases. For Policy Econ. 2022;140:102755. <https://doi.org/10.1016/j.forpol.2022.102755>
33. Wang X, Gao Q, Zou R, He X. The influence of merchant guild culture on enterprise financing behavior. Accounting Research. 2022; 168–178.
34. Woods M. Rural. London: Routledge; 2010. <https://doi.org/10.4324/9780203844304>
35. Wooldridge JM. Econometric analysis of cross section and panel data. 2nd ed. Cambridge (MA): MIT Press; 2010.
36. Wosiek M. Rural–urban divide in human capital in Poland after 1988. Oecon Copernic. 2020;11:183–201.
37. Xin S. State-driven social innovation: Can neo-exogenous development address rural marginalization? A tale of two villages in China. J Rural Stud. 2026;121:103922. <https://doi.org/10.1016/j.jrurstud.2025.103922>
38. Xu Y, Yao Y. Informal institutions, collective action, and public investment in rural China. Am Polit Sci Rev. 2015;109:371–91. <https://doi.org/10.1017/S0003055415000155>
39. Zhang X, Chen L, Liu X. Does targeted poverty alleviation foster rural entrepreneurship? Evidence from China’s township-level panel data. Econ Anal Policy. 2025;87:1927–45. <https://doi.org/10.1016/j.eap.2025.07.026>
40. Zhang Y, Zhu Q, Pan S. Fintech and individual business growth: Evidence from China. China Finance Rev Int. 2025;1–34. <https://doi.org/10.1108/CFRI-10-2024-0611>
41. Zhang Z, Liu X, Wang Y. Villages managed by the rich, institutional restriction and public goods provision: An examination of the case of irrigation in rural China. China Rural Survey. 2019;66–80.
42. Zhao T. First democracy, then centralism: The new shape of village elections under the “one-shoulder pole” policy. J Contemp China. 2024;1–15. <https://doi.org/10.1080/10670564.2024.2333010>
43. Zuo C, Huang X. Benefit or procedure? Determinants of perceived distributive fairness in rural China. World Dev. 2025;186:106821. <https://doi.org/10.1016/j.worlddev.2024.106821>
