# Supplementary material for: Businessmen-driven village governance: A viable path for rural collective economic development?
Source: PLoS One. 2026 Feb 13;21(2):e0342687. doi: 10.1371/journal.pone.0342687 (PMC12904470; doi:10.1371/journal.pone.0342687)
Supplement: Attachment — Submitted filename: Response letter.docx [file pone.0342687.s005.docx]

**Response letter**

# **Responses to Editor**

1. If possible, please upload a file showing your changes either highlighted or using track changes. This should be uploaded as a Revised Manuscript with tracked changes.

2. Can you please upload an additional copy of your revised manuscript that does not contain any tracked changes or highlighting as your main article file. This will be used in the production process if your manuscript is accepted.

3. Please include your tables as part of your main manuscript and remove the individual files.

**Response 1:**

Thank you very much for your message and for the detailed instructions. We have now uploaded the following files in accordance with your requirements:

1. A revised manuscript with all changes highlighted using track changes, uploaded as Revised Manuscript with tracked changes.
2. An additional clean version of the revised manuscript without any tracked changes or highlighting, uploaded as the main Manuscript file for production purposes.
3. All tables have been incorporated into the main manuscript file, and the individual table files have been removed.

# **Responses to Reviewer 1**

**Point 1:** Reviewer #1: All comments have been addressed

**Response 1:**

We sincerely thank Reviewer #1 for the thorough evaluation of our manuscript and for the positive assessment of our revised submission. We are grateful that the reviewer confirmed that all comments raised in the previous round have been adequately addressed and recommended the manuscript for acceptance. In the present submission, we have carefully proofread the manuscript and made minor language and formatting refinements to further improve clarity and presentation. No substantive changes have been introduced. We greatly appreciate the reviewer’s constructive feedback and support.

# **Response to Reviewer 2**

**Point 1:** The revised document satisfactorily answers all questions and addresses all concerns. The quality of the document has improved substantially and I recommend its publication.

**Response 1:**

We sincerely thank Reviewer #2 for the careful review and for the positive and encouraging evaluation of our revised manuscript. We are grateful that the reviewer confirmed that all questions and concerns have been satisfactorily addressed and recommended the manuscript for publication. In this final revision, we have focused on minor editorial improvements, including language polishing and formatting checks, while keeping all substantive content unchanged. We sincerely appreciate the reviewer’s time and valuable input.
